# PD115 Digital Health Solutions: Health Technology Assessment Of Digital Platforms For Personalized Management, Education, And Support For People With Diabetes

**DOI:** 10.1017/S0266462324003556

**Published:** 2025-01-07

**Authors:** Charlotte Bowles, Matthew Prettyjohns, Nathan Bromham, Alice Evans, Jenni Washington, Rebecca Shepherd

## Abstract

**Introduction:**

Approximately five million people live with diabetes in the UK. The cost of this is approximately 10 percent of the National Health Service (NHS) budget. Wales has the highest prevalence of diabetes of any country in the UK. Educating people on how to best manage their condition can minimize associated complications. Digital platforms can aid self-management and improve risk factors.

**Methods:**

This rapid review aimed to address the following research question: What is the clinical and cost effectiveness of digital platforms for personalized diabetes management to inform decision-making and guidance in the NHS? Digital platforms for this rapid review can be driven using artificial intelligence, machine learning, or through the application of data rules. Clinical evidence published since 2008 on health economics and patient, carer, and family perspectives relevant to Wales was identified by searching relevant databases such as MEDLINE. One relevant economic analysis was conducted using the UK Prospective Diabetes Study Outcomes Model 2.

**Results:**

Outcomes included improvements in glycemic control, healthcare resource use (e.g., number of total general practitioner and emergency department visits per year), reduction in body weight among participants, reduction in cholesterol levels, and positive patient-reported outcome measures. An economic analysis identified in the literature review found that a digital platform was more effective and less costly than routine diabetes care and was, therefore, dominant. The analysis was based on observed reductions in glycosylated hemoglobin levels from a database of people with diabetes in NHS Scotland.

**Conclusions:**

The evidence suggests there are benefits in using digital platforms to aid self-management among people with diabetes. Studies reporting on glycosylated hemoglobin levels found statistically significant and clinically important benefits from using digital platforms. Digital platforms also have the potential to be more effective and less costly than routine diabetes care in Wales and the UK.

